# Provision of Antiretroviral Therapy for HIV-Positive TB Patients — 19 Countries, Sub-Saharan Africa, 2009–2013

**Published:** 2014-11-28

**Authors:** E. Kainne Dokubo, Annabel Baddeley, Ishani Pathmanathan, William Coggin, Jacqueline Firth, Haileyesus Getahun, Jonathan Kaplan, Anand Date

**Affiliations:** 1 Division of Global HIV/AIDS, Center for Global Health, CDC; 2 Global TB Programme, World Health Organization; 3 Office of the US Global AIDS Coordinator; 4 Office of HIV/AIDS, US Agency for International Development

Considerable progress has been made in the provision of life-saving antiretroviral therapy (ART) for persons with human immunodeficiency virus (HIV) infection worldwide, resulting in an overall decrease in HIV incidence and acquired immunodeficiency syndrome (AIDS)–related mortality (1). In the strategic scale-up of HIV care and treatment programs, persons with HIV and tuberculosis (TB) are a priority population for receiving ART. TB is the leading cause of death among persons living with HIV in sub-Saharan Africa and remains a potential risk to the estimated 35 million persons living with HIV globally (1). Of the 9 million new cases of TB disease globally in 2013, an estimated 1.1 million (13%) were among persons living with HIV; of the 1.5 million deaths attributed to TB in 2013, a total of 360,000 (24%) were among persons living with HIV (2). ART reduces the incidence of HIV-associated TB disease, and early initiation of ART after the start of TB treatment reduces progression of HIV infection and death among HIV-positive TB patients (3*–*5). To assess the progress in scaling up ART provision among HIV-positive TB patients in 19 countries in sub-Saharan Africa with high TB and HIV burdens, TB and HIV data collected by the World Health Organization (WHO) were reviewed. The results found that the percentage of HIV-positive TB patients receiving ART increased from 37% in 2010 to 69% in 2013. However, many TB cases among persons who are HIV-positive go unreported (2), and only 38% of the estimated number of HIV-positive new TB patients received ART in 2013. Although progress has been made, the combination of TB and HIV continues to pose a threat to global health, particularly in sub-Saharan Africa.

Worldwide, approximately one third of persons are infected with TB. In most persons the infection is latent; however, TB can become active, infectious TB disease. HIV infection is one of the strongest risk factors for developing TB disease. To decrease the global burden of TB and HIV, WHO recommends implementation and scale-up of collaborative TB/HIV activities, including intensified TB case-finding among persons living with HIV, provider-initiated HIV testing and counseling among TB patients, and provision of ART for all HIV-positive TB patients, regardless of CD4 count. Current guidelines recommend starting TB treatment first for persons living with HIV not receiving ART at the time of TB diagnosis, then initiating ART as soon as possible within 8 weeks of TB treatment. HIV-positive TB patients with profound immunosuppression (CD4 <50 cells/*μ*L) should initiate ART within 2 weeks of starting TB treatment (6). The recommendation for universal access to ART for HIV-positive TB patients is in line with the Joint United Nations Programme on HIV/AIDS goal to have 90% of all persons with diagnosed HIV infection on ART by 2020 (7).

To assess the progress in provision of ART to HIV-positive TB patients, data were reviewed from the WHO global TB database* for 19 countries in sub-Saharan Africa with high TB and HIV burdens that are supported by the President’s Emergency Plan for AIDS Relief (PEPFAR), which supports HIV prevention, care, and treatment programs and has played a major role in the scale-up of HIV and TB services globally. A total of 6.7 million persons living with HIV are receiving ART through direct PEPFAR support, and 78% of the global TB/HIV burden is in sub-Saharan Africa (2).

From 2009 to 2013, there was an increase from 58% to 80% in the proportion of TB patients tested for HIV in the 19 PEPFAR-supported countries (Figure 1). This increase in HIV testing among TB patients in turn has led to increased detection of HIV infection. Among reported HIV-positive TB patients, there was an increase in the proportion receiving ART from 37% in 2009 to 69% in 2013 (Figure 1).

Although the proportion of HIV-positive TB patients receiving ART has increased in sub-Saharan Africa, high percentages of persons with TB disease and HIV infection are not yet receiving ART. Among reported HIV-positive TB patients in the 19 PEPFAR-supported countries, approximately 128,000 did not receive ART in 2013 (Table).

Although TB case reporting rates vary by country, the number of reported TB patients in most countries was substantially smaller than the estimated number† of TB cases because TB disease detection and reporting are incomplete (2). Consequently, ART coverage among HIV-positive TB patients is much lower when calculated using the estimated number of HIV-positive new TB patients rather than the reported number of patients (Table) (Figure 2). In the 19 countries in sub-Saharan Africa, only 38% of estimated HIV-positive new TB patients received ART in 2013 (Table).

In the PEPFAR-supported countries in sub-Saharan Africa, ART coverage in 2013 among reported TB patients who were HIV-positive ranged from 37% in Ghana to 88% in Malawi, with 17 of the 19 countries providing ART for at least 50% of HIV-positive TB patients. ART coverage based on estimated new HIV-positive TB patients ranged from a low of 9% in Nigeria to a high of 59% in Malawi, with only four of the 19 countries providing ART for at least 50% of estimated HIV-positive new TB patients.

## Discussion

ART coverage among reported HIV-positive TB patients in high TB/HIV-burden countries has risen considerably over the past several years but still falls short of the goal of 100%. The ART coverage gap is even more marked for the estimated number of new HIV-positive TB patients in sub-Saharan Africa, with only 38% ART coverage among persons in this group in PEPFAR-supported countries in 2013. Scale-up of ART provision for HIV-positive TB patients is needed to achieve the goal of 100% ART coverage for all HIV-positive TB patients, reduce morbidity and mortality of HIV-associated TB, and decrease the global burden of TB and HIV. If all estimated HIV-positive TB patients were started on ART, it would increase the number of HIV-positive persons initiating ART in 2013 by 28%, from 1.8 million to 2.3 million.

The TB and HIV syndemic continues to pose a challenge to global public health. Although there have been substantial gains in global TB control and in expansion of HIV care and treatment programs, many countries with high rates of TB and HIV have not yet reached treatment targets. To achieve the vision of an AIDS-Free Generation (8) and a world with zero TB deaths (9), concerted efforts are needed to close the ART coverage gap among all persons with TB disease and HIV infection.

Finding and diagnosing patients with TB and HIV is a prerequisite for timely TB treatment and ART initiation, and there is a survival benefit associated with early initiation of ART among profoundly immunosuppressed HIV-positive patients with TB (5). High rates of routine provider-initiated HIV testing and counseling need to be sustained for all persons with presumptive TB infection or TB disease, to enable diagnosis of HIV infection and to ensure linkage to HIV treatment programs for timely initiation of ART. HIV-positive TB patients typically access separate care and treatment systems for management of TB and HIV. When TB patients receive HIV counseling and testing and are diagnosed with HIV, they are then referred to HIV care and treatment services to be started on ART. HIV-positive TB patients can be lost to follow up because they are referred from one system to the other, and this referral process poses a challenge to patient linkage, ART initiation, and retention in HIV care and treatment. Integration of TB and HIV service delivery is critical to ensure identification of TB among persons living with HIV and diagnosis of HIV among TB patients, as well as timely TB treatment, ART initiation, and treatment adherence among HIV-positive TB patients.

TB often goes undiagnosed among persons living with HIV; therefore, intensified case-finding for TB should be a routine part of HIV care and treatment programs. Current recommendations call for persons living with HIV to be routinely screened for TB at every clinical encounter using a standard four-symptom screen of cough, fever, night sweats, and weight loss (or poor weight gain for children) at a minimum, plus TB contact history for children (6). Access to appropriate TB diagnostic tests for persons living with HIV who have TB symptoms and timely turn-around of results are critical to improving TB case detection among persons living with HIV.

Although reducing the ART gap is critical, emphasis also needs to be placed on the timing of ART provision so that treatment is initiated within 8 weeks of TB treatment for all HIV-positive TB patients, and within 2 weeks for those with CD4 <50 cells/*μ*L to achieve the mortality reduction benefits of ART. Although studies have shown an increased risk for TB immune reconstitution inflammatory syndrome with earlier initiation of ART among such cases, the risk for mortality from this syndrome has been found to be negligible (10).

The findings in this report are subject to at least two limitations. First, the proportion of HIV-positive TB patients reported to be on ART is based on national TB surveillance systems of these countries. These systems report the proportion of HIV-positive TB patients receiving ART during TB treatment and do not account for HIV-positive TB patients who might have been started on ART after completion of their TB treatment. Second, the estimated numbers of new HIV-positive TB patients used in the report are based on complex modeling conducted by WHO using multiple data sources. The effect of varying HIV testing coverage and changing HIV prevalence with increasing HIV testing coverage has not been accounted for in this model and might affect the estimates in the future.

What is already known on this topic?

Persons living with human immunodeficiency virus (HIV) are vulnerable to tuberculosis (TB), which is the leading cause of mortality among persons living with HIV worldwide. Antiretroviral therapy (ART) reduces the incidence of HIV-associated TB, reduces mortality among HIV-positive TB patients, and is recommended for all HIV-positive TB patients, regardless of CD4 count.

What is added by this report?

In 19 countries in sub-Saharan Africa with high numbers of TB patients with HIV, provision of ART for reported HIV-positive TB patients increased from 37% in 2009 to 69% in 2013. However, many TB cases among persons who are HIV-positive go unreported, and only 38% of the estimated number of HIV-positive new TB patients received ART in 2013.

What are the implications for public health practice?

The ART coverage gap of 62% of HIV-positive TB patients represents the proportion of persons with TB disease and HIV infection who should be on life-saving ART but are not yet receiving treatment. Continued scale-up of ART provision for HIV-positive TB patients is needed to reduce the morbidity and mortality of HIV-associated TB and decrease the global burden of TB and HIV.

TB and HIV data frequently come from separate TB and HIV reporting and recording systems, which are not harmonized in many settings. Improving TB and HIV monitoring and evaluation, with better integration and interoperability of TB and HIV data systems, would enhance data exchange and also improve the quality of ART coverage data for TB patients. This would facilitate early detection of treatment gaps and challenges, improve routine monitoring, inform quality improvement efforts, and help guide resource allocation to improve ART provision for HIV-positive TB patients.

## Figures and Tables

**FIGURE 1:**
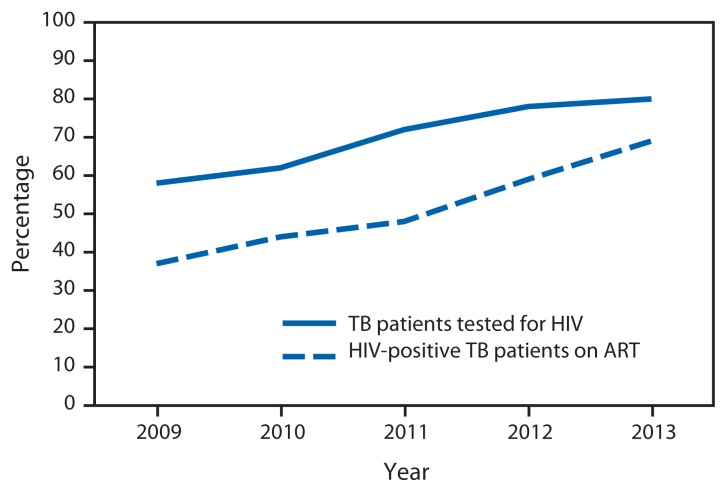
Percentages of TB patients tested for HIV and HIV-positive TB patients on antiretroviral therapy (ART) — 19 countries supported by the President’s Emergency Plan for AIDS Relief, sub-Saharan Africa, 2009–2013 **Abbreviations:** HIV = human immunodeficiency virus; TB = tuberculosis. **Source:** World Health Organization global TB database. Available at http://www.who.int/tb/country/en. ^*^ Botswana, Cameroon, Côte d’Ivoire, Democratic Republic of the Congo, Ethiopia, Ghana, Kenya, Lesotho, Malawi, Mozambique, Namibia, Nigeria, Rwanda, South Africa, Swaziland, Uganda, Tanzania, Zambia, Zimbabwe.

**FIGURE 2:**
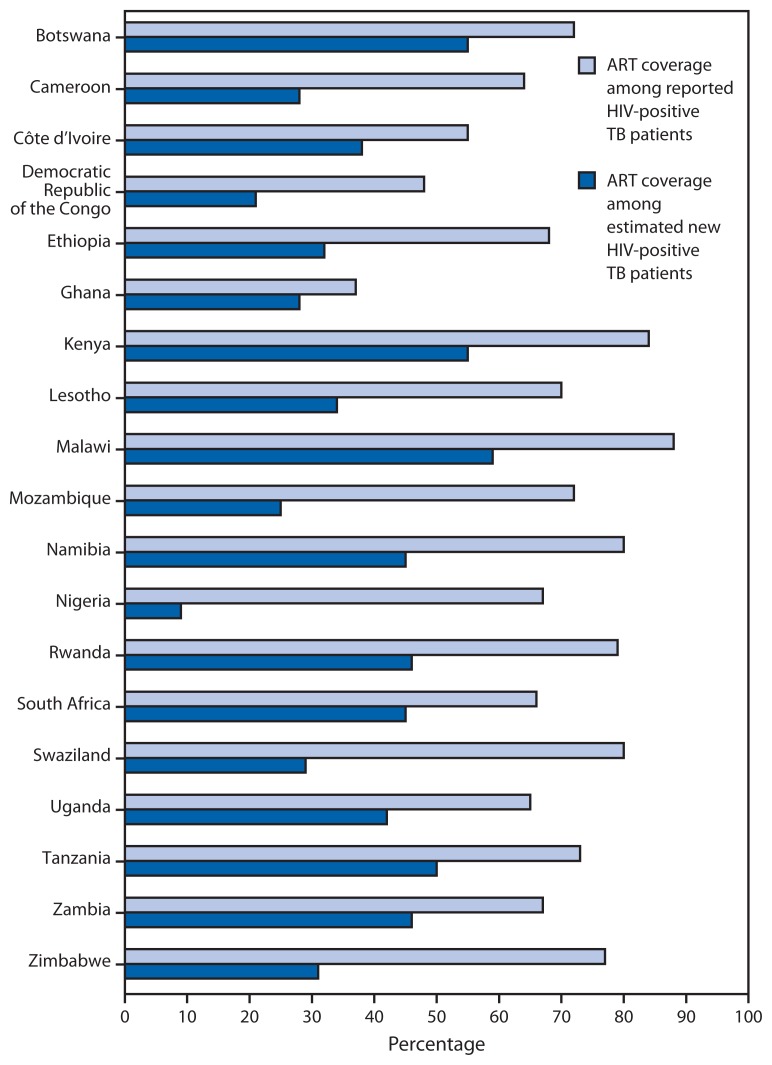
Percentage of reported HIV-positive TB patients on antiretroviral therapy (ART) compared with estimated new HIV-positive TB cases — 19 countries supported by the President’s Emergency Plan for AIDS Relief, sub-Saharan Africa, 2013 **Abbreviations:** HIV = human immunodeficiency virus; TB = tuberculosis. **Source:** World Health Organization global TB database. Available at http://www.who.int/tb/country/en.

**TABLE:** Percentage of HIV-positive TB patients on antiretroviral therapy (ART) — 19 countries supported by the President’s Emergency Plan for AIDS Relief, sub-Saharan Africa, 2013

Country	No. of reported TB patients with known HIV status	No. of TB patients who were HIV-positive	% of HIV-positive TB patients on ART	Estimated no. of new HIV-positive TB patients	% of estimated new HIV-positive TB patients on ART
Botswana	6,321	3,832	72	5,000	55
Cameroon	21,371	8,161	64	19,000	28
Côte d’Ivoire	22,502	5,506	55	8,000	38
Democratic Republic of the Congo	49,816	6,984	48	16,000	21
Ethiopia	93,356	10,374	68	22,000	32
Ghana	11,387	2,737	37	3,600	28
Kenya	84,178	31,650	84	48,000	55
Lesotho	9,756	7,234	70	15,000	34
Malawi	17,820	9,998	88	15,000	59
Mozambique	51,172	28,585	72	81,000	25
Namibia	9,727	4,343	80	7,700	45
Nigeria	88,317	19,423	67	140,000	9
Rwanda	5,882	1,447	79	2,500	46
South Africa	294,504	181,736	66	270,000	45
Swaziland	6,416	4,747	80	13,000	29
Uganda	43,318	20,648	65	32,000	42
Tanzania	54,504	20,320	73	30,000	50
Zambia	41,305	25,476	67	37,000	46
Zimbabwe	32,460	22,442	77	56,000	31
**Total**	**944,112**	**415,643**	**69**	**820,800**	**38**

**Abbreviations:** HIV = human immunodeficiency virus; TB = tuberculosis.

**Source:** World Health Organization global TB database. Available at http://www.who.int/tb/country/en.

## Footnotes

* Available at http://www.who.int/tb/country/en.

† WHO estimation methods available at http://www.who.int/tb/publications/global_report/gtbr14_online_technical_appendix.pdf?ua=1.
